# ﻿Review of the pill millipede genus *Hyperglomeris* Silvestri, 1917 (Diplopoda, Glomerida, Glomeridae) with description of two new species from Laos

**DOI:** 10.3897/zookeys.1163.103950

**Published:** 2023-05-26

**Authors:** Natdanai Likhitrakarn, Ruttapon Srisonchai, Warut Siriwut, Parin Jirapatrasilp, Ekgachai Jeratthitikul, Somsak Panha, Chirasak Sutcharit

**Affiliations:** 1 Program of Agriculture, Faculty of Agricultural Production, Maejo University, Chiang Mai 50290, Thailand Maejo University Chiang Mai Thailand; 2 Department of Biology, Faculty of Science, Khon Kaen University, Khon Kaen 40002, Thailand Khon Kaen University Khon Kaen Thailand; 3 Animal Systematics and Molecular Ecology Laboratory, Department of Biology, Faculty of Science, Mahidol University, Bangkok 10400, Thailand Mahidol University Bangkok Thailand; 4 Animal Systematics Research Unit, Department of Biology, Faculty of Science, Chulalongkorn University, Bangkok 10330, Thailand Chulalongkorn University Bangkok Thailand; 5 Academy of Science, The Royal Society of Thailand, Bangkok 10300, Thailand Academy of Science, The Royal Society of Thailand Bangkok Thailand

**Keywords:** Distribution map, key, Laos, morphology, molecular phylogeny, new species

## Abstract

The pill millipede genus *Hyperglomeris* Silvestri, 1917 is reported from Laos for the first time. Two new species, namely *H.bicaudata* Likhitrakarn, **sp. nov.** and *H.inkhavilayi* Likhitrakarn, **sp. nov.**, from Houaphanh and Khammouane provinces, northern Laos, are described and illustrated based on morphological characters and molecular analyses. Sequences of COI gene were used as DNA barcoding markers, and successfully supported the accurate identification of other Glomeridae species. Interspecific divergence of the COI uncorrected p-distance between these new species and other *Hyperglomeris* species ranged from 7.84–13.07%, while the intraspecific divergence was 0.45% in *H.inkhavilayi***sp. nov.** and 5.3% in *H.bicaudata***sp. nov.** The updated status of *Hyperglomeris*, a map of its distribution, and identification keys for all species are given.

## ﻿Introduction

The pill millipede genus *Hyperglomeris* Silvestri, 1917 belongs to the family Glomeridae, order Glomerida. Members of this family and others in the order are capable of complete volvation, where the head and collum are tucked within the rolled-up body segments or ball-like shape when threatened. For this reason they are commonly referred to as ‘pill millipedes’. Prior to this study, the genus *Hyperglomeris* consisted of only seven species, all narrowly endemic to northern Vietnam (Nguyen et al. 2019). Species of this genus are relatively small, ranging in length from 5.5 to 24 millimeters, and have a distinct body color. However, there is one colorless species, *H.depigmentata*Golovatch et al., 2013, which was found to inhabit a cave (Golovatch et al. 2013).

*Hyperglomeris* is classified within the subfamily Haploglomerinae, which has nine genera, the majority of which have only one or two species. The classification of these genera is still complicated due to few distinctive morphological characteristics, such as the presence of trichosteles on the prefemur or femur of the telopods, the number of apical cones on the antennae, and the number of striae on the thoracic shield (Wesener 2015a), and it is uncertain whether these characteristics represent species-level or genus-level distinctions. Therefore, it is essential to incorporate genetic information (as in our case, DNA barcoding based on COI gene sequences) in order to delineate species boundaries within this group. It can also be used as a foundation for further classification of the family.

Laos (or the Lao PDR) is a Southeast Asian country that shares borders with Myanmar and China to the north, Vietnam to the east, Cambodia to the south, and Thailand to the west. Laos is well-known for its beautiful mountains and forests, as well as its diverse ecosystems, which include tropical rainforests, dry lowlands, and hilly regions (ADB 2000). Previously, a total of 34 species of millipedes from 20 genera, 13 families, and seven orders have been recorded from Laos (Likhitrakarn et al. 2014). Among these, the Glomerida has remained represented by only four species of the genus *Hyleoglomeris* Verhoeff, 1910.

In this study, we were fortunate to discover two new species of the genus *Hyperglomeris* from Laos. These two new species are investigated using an approach of integrative taxonomy, combining both morphological characters and a common DNA barcoding fragment of the COI gene. In addition, we have revised the scope of the genus, also providing its distribution map and an identification key to all nine species.

## ﻿Materials and methods

### ﻿Morphological studies

Specimens were collected from Laos under the Animal Care and Use Protocol Review No. 1723018. Locations of collecting sites were recorded by GPS using a Garmin GPSMAP 60 CSx based on the WGS 84 datum, and all coordinates and elevations were double-checked with Google Earth. Photographs of live animals were taken using a Nikon 700D digital camera with a Nikon AF-S VR 105 mm macro lens. The specimens collected were euthanized by a two-step method following AVMA Guidelines for the Euthanasia of Animals (AVMA 2013). Specimens were then preserved in 90% ethanol for morphological and molecular studies. After 24 hr, ethanol was replaced with new 95% ethanol to prevent their defensive chemicals from interfering with future DNA extraction.

The holotype and all paratypes are housed in the Chulalongkorn University Museum of Zoology (**CUMZ**), Bangkok, Thailand. The specimens were examined, measured, and photographed under a Nikon SMZ 745T trinocular stereo microscope equipped with a Canon EOS 5DS R digital SLR camera. The acquired digital photos were processed and modified with Adobe Photoshop CS5. Line drawings were based on photographs taken under the stereo microscope equipped with a digital SLR camera.

The terminology used to describe morphological structures is consistent with the most recent publications (Golovatch et al. 2006; Golovatch 2017; Nguyen et al. 2019). In the catalogue sections, **D** stands for the original description; **K** for appearance in a key; **L** for appearance in a species list; **M** for a mere mention; and **MI** for molecular information. The following abbreviations listed below are used in the figures:

**cx** coxa;

**cxl** coxal lobe;

**fe** femur;

**fp** femoral process;

**NP** national park;

**pf** prefemur;

**pfc** prefemoral cone of telopod;

**pft** prefemoral trichostele of telopod;

**sh** syncoxital horn of telopod;

**sl** syncoxital lobe of telopod;

**sn** syncoxite notch;

**sx** syncoxite;

**ta** tarsus;

**tc** tibial cone;

**ti** tibia;

**tp** tibial process.

### ﻿DNA extraction, PCR amplification, and sequencing

Total genomic DNA was extracted from the legs and part of thoracic tissue of the paratype using the DNA extraction kit for animal tissue (NucleoSpin Tissue extraction kit, Macherey-Nagel, Germany), following the standard procedure of the manual. Fragments with size of 660 bp of the mitochondrial cytochrome *c* oxidase subunit I gene (COI) were amplified using LCO1490 (5’-GGTCAACAAATCATAAAGATATTGG-3’; Folmer et al. 1994) and HCOoutout (5’-GTAAATATATGRTGDGCTC; Schulmeister et al. 2002) or Nancy (5’-CCCGGTAAAATTAAAATATAAACTTC-3’; Bogdanowicz et al. 1993).

The PCR amplification was performed using a T100 thermal cycler (BIO-RAD) with a final reaction volume of 20 μL (15 μL of EmeraldAmp GT PCR Master Mix, 1.5 μL of each primer, 10 ng of template DNA and distilled water up to 20 μL total volume). Thermal cycling was performed at 94 °C for 3 min, followed by 35 cycles of 94 °C for 30 s, annealing at 42–56 °C (depending on samples and the primer pair) for 60 s, extension at 72 °C for 90 s, and a final extension at 72 °C for 5 min. Amplification of PCR products was confirmed through 1.5% (w/v) agarose gel electrophoresis before purification by MEGAquick-spinTM plus (Fragment DNA purification kit) and sequencing in both directions (forward and reverse) using an automated sequencer (ABI prism 3730XL).

All nucleotide sequences obtained in this study were deposited in the GenBank Nucleotide sequences database under accession numbers OQ661871–OQ661874. The collecting localities and GenBank accession numbers of each nominal species are listed in Table 1.

**Table 1.** List of species used for molecular phylogenetic analyses and relevant information. * = paratype.

Voucher number	Species	Locality	Geographical coordinates	GenBank accession number COI	Reference
CUMZ-GLO004*	*Hyperglomerisbicaudata* Likhitrakarn, sp. nov.	Ban Tham Na Tan, Houaphanh, Laos	20°27'28"N, 104°08'43"E	OQ661871	This study
CUMZ-GLO007*	*Hyperglomerisbicaudata * Likhitrakarn, sp. nov.	Limestone mountain area near vocational-technical school, Houaphanh, Laos	20°24'15"N, 104°15'4"E	OQ661872	This study
CUMZ-GLO095/1*	*Hyperglomerisinkhavilayi* Likhitrakarn, sp. nov.	Orchard, Ban Phawong, Khammouane, Laos	17°32'24"N, 105°25'18"E	OQ661873	This study
CUMZ-GLO095/2*	*Hyperglomerisinkhavilayi* Likhitrakarn, sp. nov.	Orchard, Ban Phawong, Khammouane, Laos	17°32'24"N, 105°25'18"E	OQ661874	This study
FMNH-SVE102	*Hyperglomerissimplex* Nguyen et al., 2019	Tam Dao NP, Vinh Phuc, Vietnam		MT749410	Nguyen et al. 2021
IEBR-Myr 605	*Hyperglomerissimplex* Nguyen et al., 2019	Tam Dao NP, Vinh Phuc, Vietnam		MT749403	Nguyen et al. 2021
IEBR-Myr 674	*Hyperglomeris* sp. 1	Pu Mat NP, Nghe An, Vietnam		MT749409	Nguyen et al. 2021
FMNH-SVE176	*Peplomerismagna* Golovatch, 1983	Cuc Phuong NP, Ninh Binh, Vietnam		MT749407	Nguyen et al. 2021
IEBR-Myr 677	*Peplomerismagna* Golovatch, 1983	Cuc Phuong NP, Ninh Binh, Vietnam		MT749405	Nguyen et al. 2021
IEBR-Myr 656	*Peplomerismagna* Golovatch, 1983	Cuc Phuong NP, Ninh Binh, Vietnam		MT749408	Nguyen et al. 2021
FMNH-SVE204	*Hyleoglomerislobus* Nguyen et al., 2019	Cuc Phuong NP, Ninh Binh, Vietnam		MT749391	Nguyen et al. 2021
IEBR-Myr 653	*Hyleoglomerislobus* Nguyen et al., 2019	Cuc Phuong NP, Ninh Binh, Vietnam		MT749402	Nguyen et al. 2021
IEBR-Myr 678	*Hyleoglomerislobus* Nguyen et al., 2019	Cuc Phuong NP, Ninh Binh, Vietnam		MT749406	Nguyen et al. 2021
IEBR-Myr 533	*Rhopalomerissauda* Nguyen et al., 2019	Ba Be NP, Bac Kan, Vietnam		MT749404	Nguyen et al. 2021
IEBR-Myr 654	*Rhopalomerissauda* Nguyen et al., 2019	Me Linh District, Vinh Phuc, Vietnam		MT749401	Nguyen et al. 2021
IEBR-Myr 706	*Rhopalomerissauda* Nguyen et al., 2019	Khao Ca NR, Ha Giang, Vietnam		MT749400	Nguyen et al. 2021
IEBR-Myr 801	*Rhopalomerissauda* Nguyen et al., 2019	Cham Chu NR, Tuyen Giang, Vietnam		MT749398	Nguyen et al. 2021
IEBR-Myr 804a	*Tonkinomerisnapoensis* Nguyen et al., 2019	Bac Me NR, Ha Giang, Vietnam		MT749397	Nguyen et al. 2021
IEBR-Myr 804b	*Tonkinomerisnapoensis* Nguyen et al., 2019	Bac Me NR, Ha Giang, Vietnam		MT749396	Nguyen et al. 2021
Tcost8-MK	*Trachysphaeracostata* (Waga, 1857)	Slovakia		KX467622	Mock et al. 2016
GBOL33714	*Glomerismarginata* (Villers, 1789)	Königshütte, Wernigerode, Sachsen-Anhalt, Germany	51.743°N, 10.767°E	MG892112	Reip and Wesener 2018
ZFMK1634	*Glomerismarginata* (Villers, 1789)	Bockswiese Goslar, Niedersachsen, Germany	51.841°N, 10.326°E	MG892119	Reip and Wesener 2018
**Outgroup Sphaerotheriida: Zephroniidae**					
FMNH-INS 0000 072 674)	*Sphaerobelumtruncatum* Wongthamwanich, 2012	Pang Hi Village, Nan, Thailand	19°23'46.3"N, 100°41'42.4"E	JN885184	Wongthamwanich et al. 2012
ZFMK Myr3502	*Zephronialaotica* Wesener, 2019	Garden of Erawan Riverside Hotel, Champasak, Laos	15°6'27.0"N, 105°49'14.3"E	MK330977	Wesener 2019

### ﻿Phylogenetic analyses

The sequences were aligned using MEGA7 (Kumar et al. 2016). The final aligned dataset included 660 bp of 23 COI sequences. All species of *Hyperglomeris* for which sequences are available in Genbank were included, along with members of some other genera of Glomeridae (i.e., *Peplomeris* Silvestri, 1917, *Tonkinomeris* Nguyen, Sierwald & Marek, 2019, *Rhopalomeris* Verhoeff, 1906, *Hyleoglomeris*, *Glomeris* Latreille, 1802, and *Trachysphaera* Heller, 1857); the genera *Sphaerobelum* and *Zephronia* were used as outgroups (Table 1).

Two phylogenetic methods were used in this study. Firstly, the maximum likelihood (ML) method was performed using RAxML v. 8.2.10 (Stamatakis, 2014), with GTRGAMMA as the nucleotide substitution model and 1,000 ML bootstrap replicates to assess topology bootstrap support (bp). Secondly, Bayesian Inference (BI) analysis was performed by MrBayes 3.2.6 (Ronquist et al. 2012) using the Markov chain Monte Carlo technique (MCMC), and with the best-fit model of nucleotide substitution as suggested by PartitionFinder2 v. 2.3.4 (Lanfear et al. 2016). The selected best-fit models for the three COI codon positions were SYM+G, F81+I, and GTR+G, respectively. The BI trees were run for ten million generations with a random starting tree. The resultant trees were sampled every 1,000 generations and the values were used to estimate consensus tree topology; bipartition posterior probability (bpp), and branch lengths, after the first 25% of obtained trees were discarded as burn-in. All average effective sample size (ESS) values sampled from the MCMC analysis were greater than 2,000 in all parameters. Both phylogenetic analyses were implemented through the on-line CIPRES Science Gateway (Miller et al. 2010). The obtained tree was drawn by FigTree v. 1.4.3 (http://tree.bio.ed.ac.uk/software/figtree/, accessed on 28 February 2023). In addition, genetic divergence based on the COI sequence was evaluated using uncorrected p-distances as implemented in MEGA7 (Kumar et al. 2016). The missing data in pairwise comparison were treated with pairwise deletion method.

## ﻿Taxonomy

### ﻿Family Glomeridae Leach, 1815

#### ﻿Genus Hyperglomeris

Taxon classificationAnimaliaGlomeridaGlomeridae

Silvestri, 1917

F79E0EAD-16FA-589D-8A77-028F00033DAD

Hyperglomeris Silvestri, 1917: 145 (D, K).
Hyperglomeris –Golovatch, 1983a: 110 (M); 2017: 196 (M); Golovatch et al. 2013: 202 (M); Nguyen et al. 2019: 274 (M, K); 2021: 257 (M); Kuroda et al. 2022a: 162 (M); 2022b: 117 (M).
Dinoglomeris Silvestri, 1917: 147 (D, K), synonymized by Golovatch (1983b: 180).

##### Diagnosis.

Pill millipedes with four apical cones on the antennae; the caudal margins of the pygidium are sometimes modified into small paramedian lobes, but are mostly emarginate or slightly concave medially; leg-pair 18 devoid of any evident mesal outgrowths on the femur or tibia; the posterior telopods are rather stout, with prefemoral trichosteles reduced or only present as a small cone; and the femoral trichosteles are strongly reduced or absent.

##### Type species.

*Hyperglomerislamellosa* Silvestri, 1917, by original designation.

##### Species included.

*Hyperglomerislamellosa* Silvestri, 1917, *H.dirupta* (Silvestri, 1917), *H.conspicua* Golovatch, 1983, *H.maxima* Golovatch, 1983, *H.depigmentata* Golovatch, Geoffroy & VandenSpiegel, 2013, *H.nigra* Golovatch, 2017, *H.simplex* Nguyen, Sierwald & Marek, 2019, *H.bicaudata* Likhitrakarn, sp. nov., *H.inkhavilayi* Likhitrakarn, sp. nov.

##### Remarks.

The genus *Hyperglomeris* was established by Silvestri (1917), who designated *H.lamellosa* Silvestri, 1917 as the type species and provided a detailed description and excellent illustrations. At the same time, he created a new genus and species, *Dinoglomerisdirupta*, which only superficially differed from *Hyperglomeris*. Both species were discovered on Mount Mẫu Sơn, Vietnam, but at different altitudes. Subsequently, Golovatch (1983b) investigated the scope of the genus using his material from Vietnam and proposed that the two genera be combined into one. Afterwards, Golovatch published two new species, synonymizing the name *Dinoglomeris* with *Hyperglomeris*.

#### ﻿ Hyperglomeris lamellosa

Taxon classificationAnimaliaGlomeridaGlomeridae

Silvestri, 1917

6BE3C4A3-75CC-54CE-9E61-2C6011ED072E

Hyperglomeris lamellosa Silvestri, 1917: 147 (D); Golovatch 1983a: 110 (M, K); 1983b: 180 (L); 2017: 196 (M, K); Golovatch et al. 2013: 201 (M); Enghoff et al. 2004: 31 (L); Nguyen et al. 2019: 263 (L, M).

##### Remarks.

This species was described from Mount Mẫu Sơn, 2000–3000 feet a.s.l., Langson Province, Vietnam (Silvestri 1917). Endemic to Vietnam.

#### ﻿ Hyperglomeris dirupta

Taxon classificationAnimaliaGlomeridaGlomeridae

(Silvestri, 1917)

6C86D218-17CA-5873-A562-2E5C6C4B85CB

Dinoglomeris dirupta Silvestri, 1917: 147 (D).
Hyperglomeris dirupta –Golovatch, 1983a: 110 (M, K); 1983b: 180 (L); 2017: 196 (M, K); Golovatch et al. 2013: 201 (M); Enghoff et al. 2004: 31 (L); Nguyen et al. 2019: 263 (L, M).

##### Remarks.

This species was described from Mount Mẫu Sơn, 200–300 feet a.s.l., Langson Province, Vietnam (Silvestri 1917). Endemic to Vietnam.

#### ﻿ Hyperglomeris conspicua

Taxon classificationAnimaliaGlomeridaGlomeridae

Golovatch, 1983

197BD0A0-8EC9-5F17-8DA7-D4D63AC559FA

Hyperglomeris conspicua Golovatch, 1983a: 110 (D, K); Golovatch 1983b: 180 (L); 2017: 197 (M, K); Golovatch et al. 2013: 201 (M); Enghoff et al. 2004: 31 (L); Nguyen et al. 2019: 262 (L, M).

##### Remarks.

This species was described from Vạn Mai, Mai Châu District, Hòa Bình Province, Vietnam (Golovatch 1983a). Endemic to Vietnam.

#### ﻿ Hyperglomeris maxima

Taxon classificationAnimaliaGlomeridaGlomeridae

Golovatch, 1983

6032232A-4112-5944-803A-D76BBE854FF7

Hyperglomeris maxima Golovatch, 1983a: 108 (D, K); Golovatch, 1983b: 180 (L); 2017: 197 (M, K); Golovatch et al. 2013: 201 (M); Enghoff et al. 2004: 31 (L); Nguyen et al. 2019: 263 (L, M).

##### Remarks.

This species was described from Vạn Mai, Mai Châu District, Hòa Bình Province, Vietnam (Golovatch 1983a). Endemic to Vietnam.

#### ﻿ Hyperglomeris depigmentata

Taxon classificationAnimaliaGlomeridaGlomeridae

Golovatch, Geoffroy & VandenSpiegel, 2013

7F67E6AB-8F3C-55B9-902A-0BEFC69343D9

Hyperglomeris depigmentata Golovatch et al., 2013: 206 (D); Golovatch 2017: 197 (M, K); Nguyen et al. 2019: 262 (L, M); Kuroda et al. 2022a: 162 (M, K).

##### Remarks.

This species was described from Cave Hang Doi, 20.496176°N, 105.137465°E, Lang Kho Muong, Than Son, Thanh Hoa Province, Vietnam (Golovatch et al. 2013). Endemic to Vietnam.

#### ﻿ Hyperglomeris nigra

Taxon classificationAnimaliaGlomeridaGlomeridae

Golovatch, 2017

B9D365F3-605E-549F-9879-2EEA3E05CA1B

Hyperglomeris nigra Golovatch, 2017: 195 (D, K); Nguyen et al. 2019: 263 (L, M).

##### Remark.

This species was described from Xuan Son National Park, 21°07'52"N, 104°57'07"E, 400–470 m a.s.l., ca. 90 km northwest of Hanoi, Phu Tho Province, Vietnam (Golovatch 2017).

#### ﻿ Hyperglomeris simplex

Taxon classificationAnimaliaGlomeridaGlomeridae

Nguyen, Sierwald & Marek, 2019

3C2B2E0C-7A8D-59A9-9302-2ABD2BC66530

Hyperglomeris simplex Nguyen et al., 2019: 276 (D).
Hyperglomeris simplis (sic!)–Nguyen et al. 2021: 258 (MI, M).

##### Remark.

This species was described from Me Linh Station for Biodiversity, 21.3850°N, 105.7119°E, Ngoc Thanh Commune, Phuc Yen Town, Vinh Phuc Province, Vietnam (Nguyen et al. 2019).

#### ﻿ Hyperglomeris bicaudata

Taxon classificationAnimaliaGlomeridaGlomeridae

Likhitrakarn sp. nov.

0D9D3124-A7FA-53E2-A716-3DC07AB1A65B

https://zoobank.org/FB13C74A-496A-45F0-BB73-C710A6C5123D

Figs 1 , 2 , 3A, B

##### Material examined.

***Holotype*: Laos – Houaphanh** • ♂ (CUMZ-GLO006); Viengxay District, Limestone mountain area near Kaysone Phomvihane Cave; elev. 890 m a.s.l.; 20°20'24"N, 104°13'44"E; 6 Jul. 2014; R. Srisonchai, C. Sutcharit, K. Inkhavilay leg.; CUMZ; ***Paratypes*: Laos** – **Houaphanh** • 1 ♀; same collection data as holotype; • 3 ♀♀ (CUMZ-GLO004); Viengxay District, Ban Tham Na Tan, Limestone mountain area; elev. 860 m a.s.l.; 20°27'28"N, 104°08'43"E; 5 Jul. 2014; R. Srisonchai, C. Sutcharit, K. Inkhavilay leg.; CUMZ; OQ661871 • 1 ♂, 2 ♀♀ (CUMZ-GLO007); Viengxay District, Limestone mountain area near vocational-technical school around kilometre 31; elev. 840 m a.s.l.; 20°24'15"N, 104°15'4"E; 6 Jul. 2014; R. Srisonchai, C. Sutcharit, K. Inkhavilay leg.; CUMZ; OQ661872.

##### Name.

To emphasize the caudal margin of the anal shield being more (♂) or less (♀) strongly bisinuate medially; adjective in feminine gender.

##### Diagnosis.

Its unique color pattern is similar to that of *H.nigra* Golovatch, 2017, from Vietnam (Golovatch, 2017), but the two species differ by the thickness of the contrasting paler bands at the lateral and caudal edges of all tergites (ca. 1/3 vs. 1/5× as high as tergite height), the number of striae at the lateral edge of midbody tergites (2 vs. 3), the number of ommatidia (10+1(2) vs. 8+1), coupled with two tibial processes (one large process and one small cone vs. two small tibial cones), and the caudal edge of the anal shield (two strongly bisinuate medially vs. slightly emarginate medially).

##### Description.

***Body length*** of stretched holotype 13.2 mm, width 8.3 mm. Body length of stretched paratypes 13.5 mm (♂), 13.5–15.5 mm (♀), width 9.5 (♂), 8.5–9.5 mm (♀).

**Figure 1.**
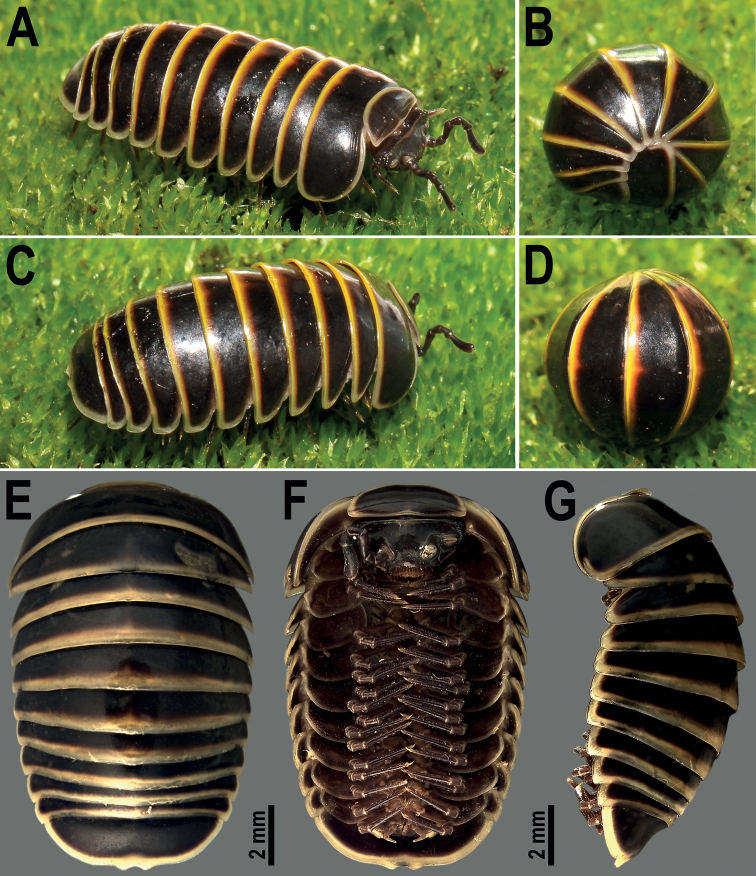
*Hyperglomerisbicaudata* sp. nov. **A–D** ♂ paratype (CUMZ-GLO006), habitus, live coloration **E–G** ♂ holotype (CUMZ-GLO006) habitus in dorsal, ventral, and lateral views **A, C** unrolled, sublateral views **B, D** rolled, sublateral and subdorsal views, respectively. **A–D** not to scale.

***Coloration of live animals*** (Fig. 1A–D): body blackish, with contrasting pale yellow to orange yellow, rather broad bands at the lateral and caudal edges of all tergites, ca. 1/3× as high as each tergite height, including collum, thoracic and anal shields. Head and antennae black, only labrum and Tömösváry’s organ yellowish. Venter and legs dark brown to brown with a pale yellowish claw and the posterior part of each tarsus; coloration in alcohol faded after eight years of preservation (Fig. 1E–G), body pale black to charcoal, with contrasting pale yellow to whitish bands. Head and antennae grey to blackish. Venter and legs pale brown to brownish.

***Labrum sparsely setose*** (Fig. 1F). Gnathochilarium with 2+2 palps of subequal length. Ocular fields whitish, 10+1(2) ommatidia, cornea convex, oval in shape, translucent. Antennae with four evident apical cones, segment 6 ca. 2.1–2.4× as long as high. Organ of Tömösváry typical, horseshoe-shaped, oblong-oval, elongate, ca. 1.5–1.8× as long as broad (Fig. 1F).

**Figure 2.**
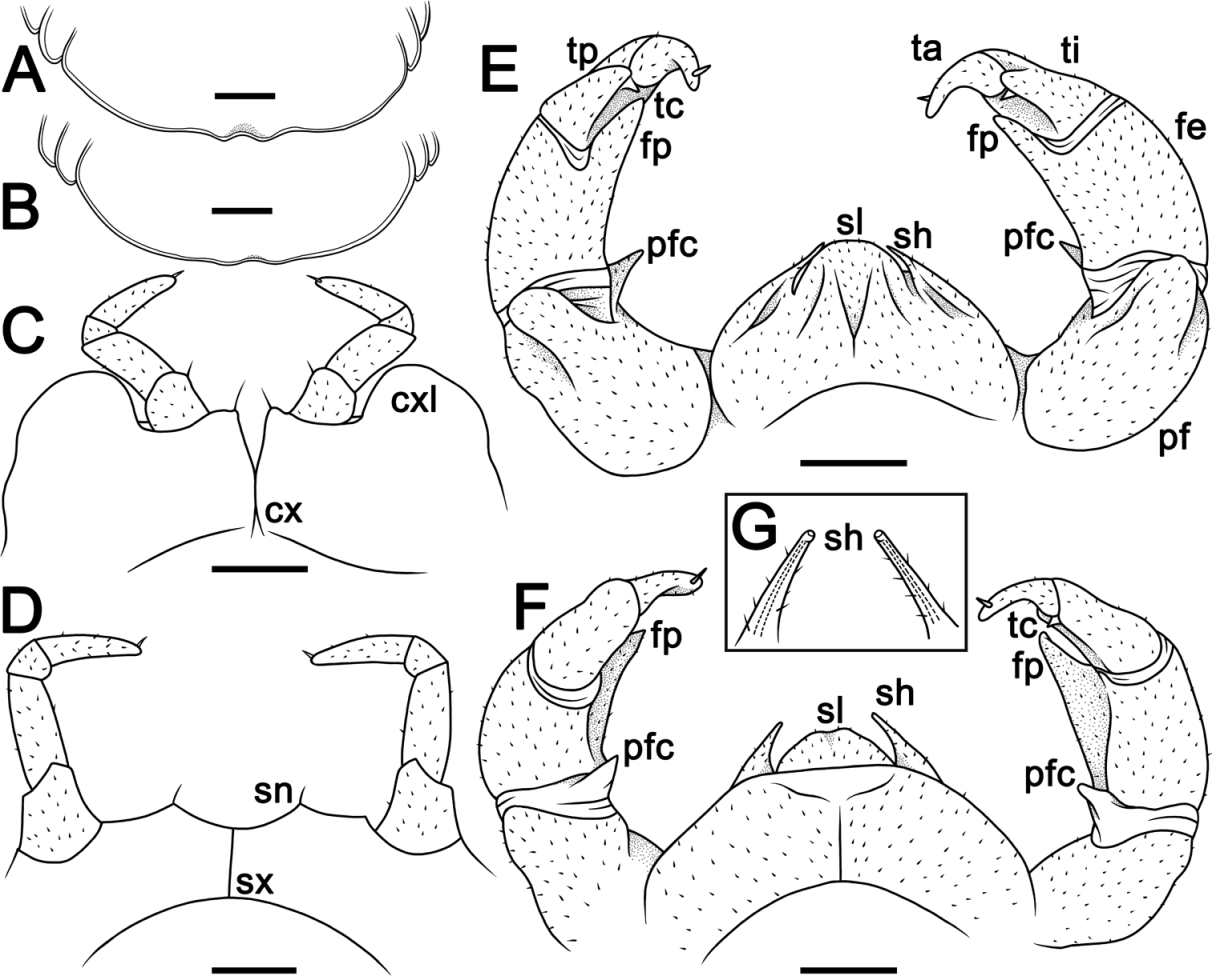
*Hyperglomerisbicaudata* sp. nov. **A, C, E, F** ♂ holotype (CUMZ-GLO006) **B** ♀ paratype (CUMZ-GLO006) **D** ♂ paratype (CUMZ-GLO007) **A, B** anal shield edge in venter view, male and female, respectively **C** leg 17, anterior view **D** leg 18, anterior view **E, F** telopod, posterior and anterior views, respectively **G** tip of syncoxital lobes (not to scale). Scale bars: 1 mm (**A–F)**. Abbreviations: **cx** coxa, **cxl** coxal lobe, **fe** femur, **fp** femoral process, **pf** prefemur, **pfc** prefemoral cone of telopod, **sh** syncoxital horn of telopod, **sl** syncoxital lobe of telopod, **sn** syncoxite notch, **sx** syncoxite, **ta** tarsus, **tc** tibial cone, **ti** tibia, **tp** tibial process.

***Collum*** as usual, with two transverse striae (Fig. 1F). Thoracic shield with a small hyposchism field not projecting caudad past tergal margin. Striae 4–6, mostly superficial, only lower 3 or 4 lying above schism, one level with schism, remaining 1 or 2 below schism, with 4 and 5 complete, crossing the dorsum (Fig. 1G). Terga 3 and 4 rather broadly rounded laterally (Fig. 1G). Following terga in front of pygidium faintly concave medially at caudal edge and with two striae starting above lateral edge, sometimes first stria fading away towards midway. Caudal edge of anal shield more (♂, Figs 1C, E, F, G, 2A) or less (♀, Fig. 2B)) strongly bisinuate medially.

***Male legs 17*** (Fig. 2C) strongly reduced, with a rather high, often irregularly rounded coxal lobe (cxl) and a 4-segmented telopodite.

**Figure 3.**
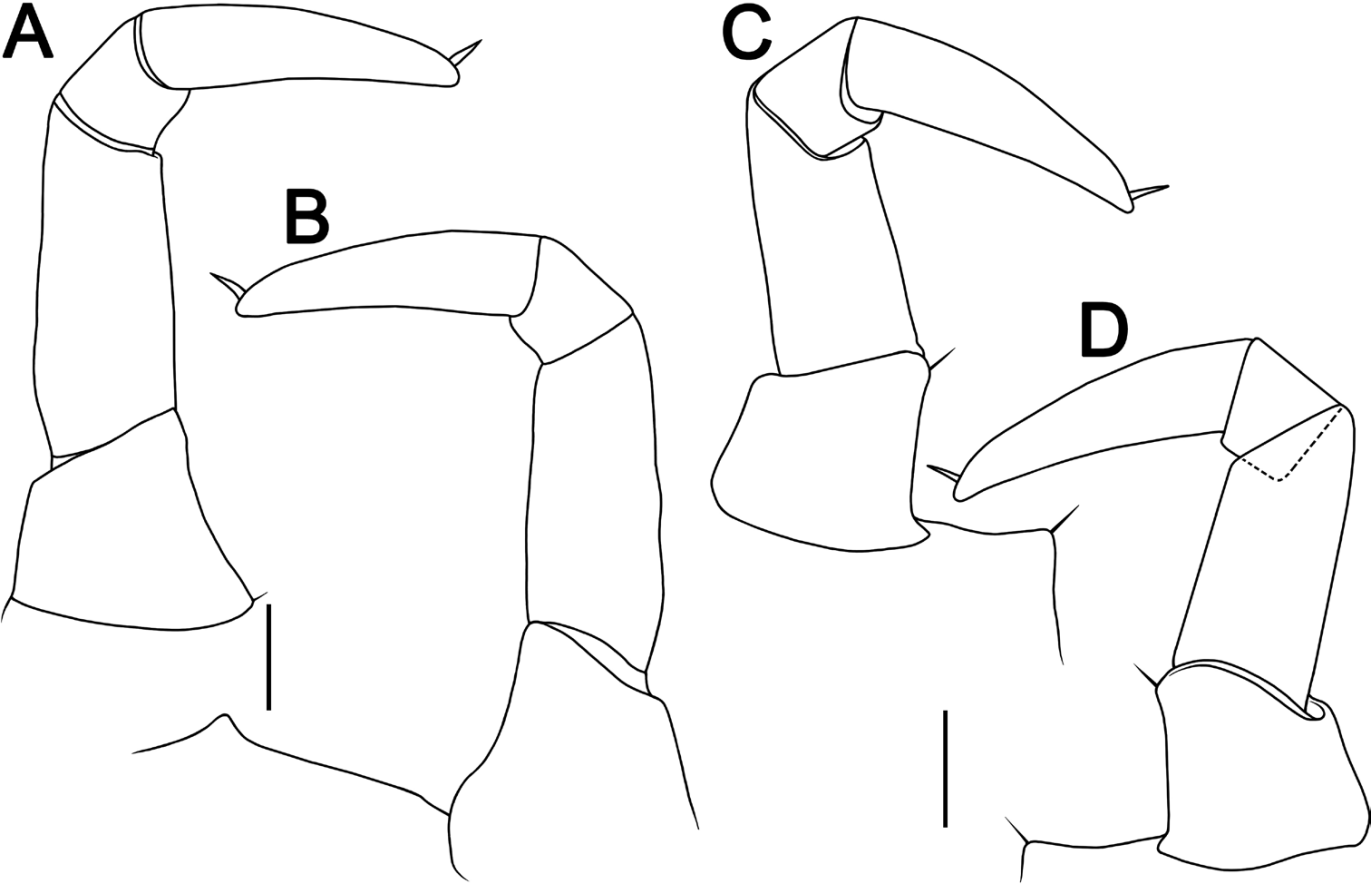
Leg 18 **A, B***Hyperglomerisbicaudata* sp. nov., ♂ paratype (CUMZ-GLO007), left, anterior and posterior views, respectively **C, D***Hyperglomerisinkhavilayi* sp. nov., ♂ paratype, right, anterior, and posterior views, respectively. Scale bars: 1 mm.

***Male legs 18*** (Figs 2D, 3A, B) simple, rather strongly reduced, without any evident outgrowths; syncoxite membranous, on either side with a simple, small, and narrowly ogival syncoxite notch (sn) and a 4-segmented telopodite.

***Telopods*** (= male legs 19) (Fig. 2E–G) with a very large, broad and roundly subtrapeziform syncoxital lobe (sl) flanked by two short, spiniform, obliquely truncate, setose syncoxital horns (sh), level with syncoxital lobe (Fig. 2F). Telopodite 4-segmented, with a spine apically. Prefemur subellipsoid, with an evident, rather small, distad tapering, tuberculiform, distomesal prefemoral cone (pc) (a reduced trichostele), ca. 1/4–1/5× as long as femur. The latter in caudal view with a prominent, stout, finger-shaped, distomesal femoral process (fp) devoid of a trichostele, produced apically to ca. 3/4 tibia. Tibia elongate, gently tapering distad and curved apically basad towards process on femur, with an evident, caudad curved, distolateral tibial process (tp) and a small, short and pointed distomesal tibial cone (tc). Tarsus smallest, subcylindrical, moderately sigmoid, strongly curved, narrowly rounded apically.

##### Remarks.

Unique to this species is that the caudal margin of the anal shield shows two more (♂, Figs 1C, E, F, G, 2A) or less (♀, Fig. 2A) pronounced paramedian knobs. That the male is equipped with such modifications is quite usual in various lineages of Glomerida (e.g., Liu and Golovatch 2020), but their presence in the female, albeit not as strongly as in the male, is really striking.

This distinguishing character can be hypothesized as possibly playing an important role in a courtship process or being associated with courtship behavior. Certain male structures dedicated to interactions with females during courtship have often diverged relatively quickly during evolution, causing these features to change into species-specific differences (Eberhard 2004). Noteworthy examples of such characters are antennae, legs and heads in springtails (Collembola: Bourletiellidae) (Kozlowski and Aoxiang 2006) and stridulation organs in giant pill millipedes (Sphaerotheria) (Wesener et al. 2011) that may not be involved directly in sperm transfer but are associated with mating behavior. In order to understand the relationship between these types of traits and their function in the glomerids, it is essential to examine the mating behavior of this species.

#### ﻿ Hyperglomeris inkhavilayi

Taxon classificationAnimaliaGlomeridaGlomeridae

Likhitrakarn sp. nov.

5A1AAAC4-7BB3-5E5A-A37F-7E6B766E8E09

https://zoobank.org/60149C6A-59F8-4AD9-82EC-7A8A1CCBD7EA

Figs 3C, D , 4 , 5

##### Material examined.

***Holotype*: Laos – Khammouane** • ♂ (CUMZ-GLO095); Nhommalath District, Ban Phawong, orchard; elev. 190 m a.s.l.; 17°32'24"N, 105°25'18"E; 25 Aug. 2014; R. Srisonchai, C. Sutcharit, K. Inkhavilay leg.; CUMZ; ***Paratypes*: Laos** – **Khammouane** • 2 ♂♂, 3 ♀♀; same collection data as holotype; OQ661873, OQ661874.

##### Name.

To honor Dr. Khamla Inkhavilay, the director of the Center of Excellence in Biodiversity at National University of Laos, Vientiane, Laos, who participated in collecting the type series.

##### Diagnosis.

Although its color pattern seems to be similar to that of *H.simplex* Nguyen, Sierwald & Marek, 2019 (Nguyen et al. 2019), it differs by the coloration of the collum, thoracic and anal shields (mostly pale yellowish to brownish vs. dark brown to blackish), leg-pair 18 (with a simple, subtriangular syncoxital notch (sn) vs. an evident pronounced syncoxital tubercles), coupled with a longer prefemoral trichostele (pt) (more than 2/3 vs. 1/2 of femur).

##### Description.

***Body length*** of stretched holotype 10.7 mm, width 6.9 mm. Body length of stretched paratypes 9.3 mm (♂), 13.5–15.5 mm (♀), width 5.6 (♂), 5.2–8.5 mm (♀).

**Figure 4.**
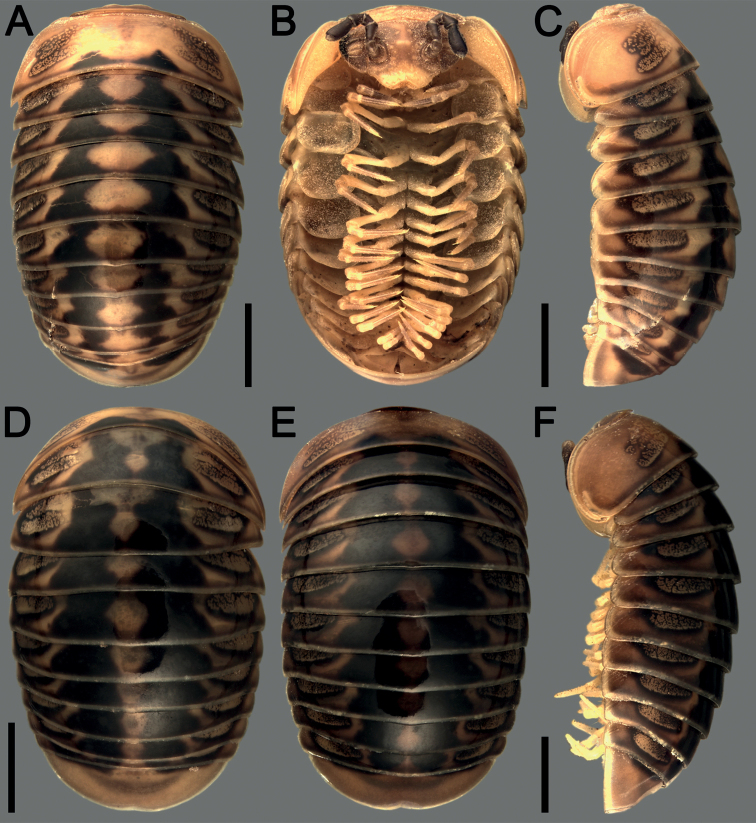
*Hyperglomerisinkhavilayi* sp. nov., habitus in dorsal, ventral, and lateral views **A–C** ♀ paratype (CUMZ-GLO095) **D** male paratype (CUMZ-P085) **E, F** ♂ holotype (CUMZ-P085). Scale bars: 2 mm.

***Coloration*** in alcohol faded after eight years of preservation (Fig. 4), body mostly yellowish, with contrasting black paramedian spots flanking the midline. Mid-dorsal spots on each of tergites 3–11 usually subtriangular (Fig. 4A, D), or parallel-sided (Fig. 4E), with smaller and detached patches at caudal edge of tergite 2 and at anterior edge of tergite 12. Lateral sides of each of tergites 2–11 also with a pair of large, sublateral, yellow to marbled blackish spots beside the triangles, normally not reaching the translucent caudal and lateral edges (Fig. 4A, C–E). Head and collum pale yellowish to dark brownish with darker color laterally. Antennae black to dark brown, only tip of antennae yellowish. Legs and venter pale yellowish to pale brown (Fig. 4B).

***Labrum sparsely setose*** (Fig. 4B). Gnathochilarium with 2+2 palps of subequal length. Ocular fields blackish, 8(7)+1 ommatidia, cornea very convex, translucent. Antennae with four evident apical cones, segment 6 ca. 2.1–2.4× as long as high (Fig. 4B). Organ of Tömösváry typical, horseshoe-shaped, oblong-oval, elongate, ca. 1.3–1.5× as long as broad (Fig. 4B).

***Collum*** as usual, with two transverse striae. Thoracic shield with a small hyposchism field not projecting caudad to nearly reaching the tergal margin. Striae 5–7, mostly superficial, only lower 2 or 3 lying above schism, one level with schism, remaining 3 or 4 below schism, with 5 or 6 complete, crossing the dorsum. Terga 3 and 4 broadly rounded laterally (Fig. 4C, F). Following terga in front of anal shield rather clearly concave medially at caudal edge and with 2–4 striae starting above lateral edge. Male anal shield slightly concave medially at caudal edge (Fig. 4D, E).

**Figure 5.**
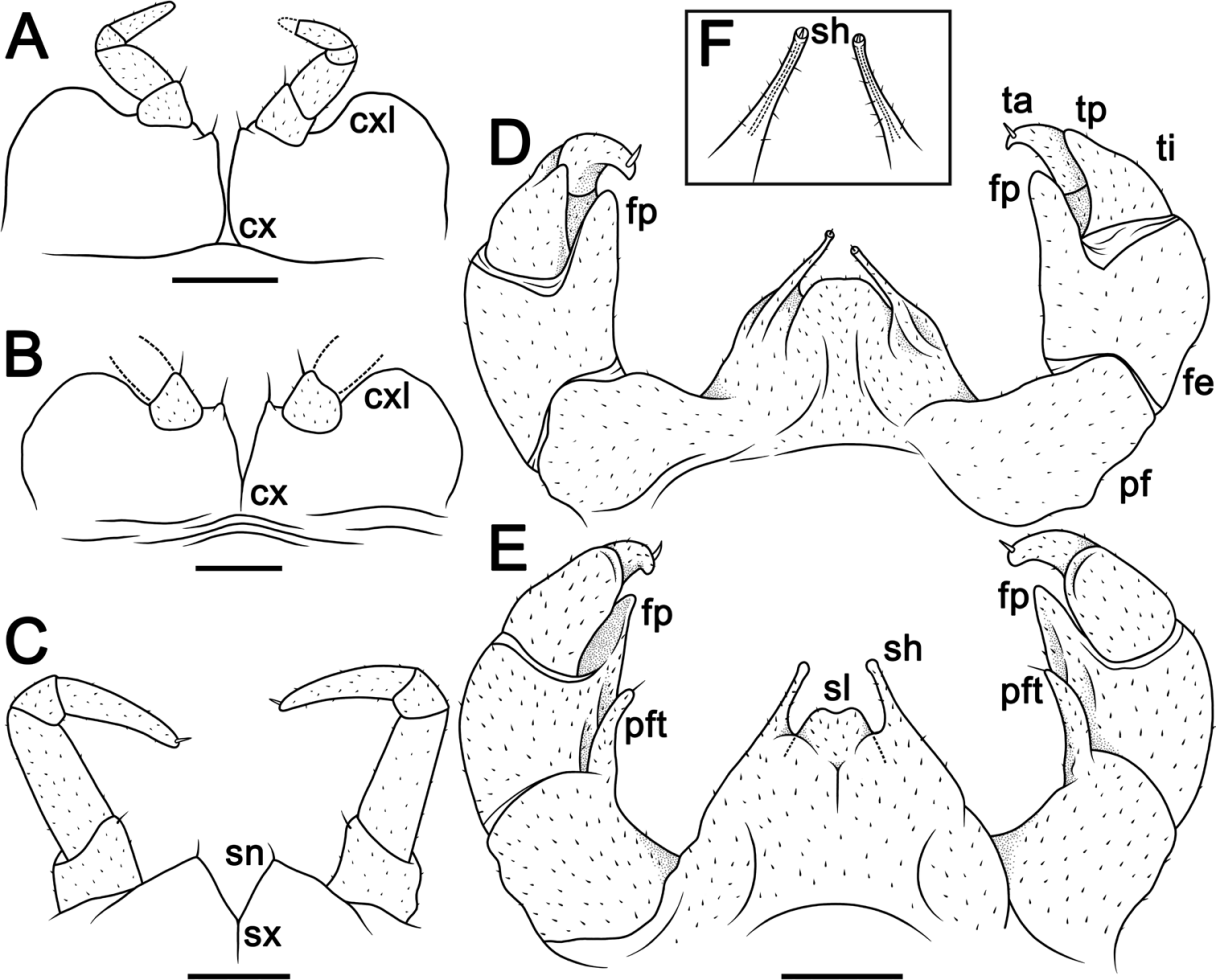
*Hyperglomerisinkhavilayi* sp. nov. **A** ♂ paratype (CUMZ-GLO095) **B–F** ♂ holotype (CUMZ-P085) **A, B** leg 17, anterior views **C** leg 18, anterior view **D, E** telopod, posterior and anterior views **F** tip of syncoxital lobes (not to scale). Scale bars: 1 mm (**A–E**). Abbreviations: **cx** coxa, **cxl** coxal lobe, **fe** femur, **fp** femoral process, **pf** prefemur, **pft** prefemoral trichostele of telopod, **sh** syncoxital horn of telopod, **sl** syncoxital lobe of telopod, **sn** syncoxite notch, **sx** syncoxite, **ta** tarsus, **tc** tibial cone, **ti** tibia, **tp** tibial process.

***Male legs 17*** (Fig. 5A, B) strongly reduced, with a rather large, often irregularly rounded coxal lobe (cxl) and a 4-segmented telopodite.

***Male legs 18*** (Figs 3C, D, 5C) simple, rather strongly reduced, without any evident outgrowths; syncoxite membranous, on either side with a broad, subtriangular syncoxital notch (sn) and a 4-segmented telopodite.

***Telopods*** (= male legs 19) (Fig. 5D–F) with a large, rather roundly subtrapeziform syncoxital lobe (sl) with a small notch medially (Fig. 5E), flanked by two very long, spiniform, obliquely truncate, setose syncoxital horns (sh), two × as high as syncoxital lobe (Fig. 5D, E). Telopodite 4-segmented, with a spine apically. Prefemur subquadrangular, with a long, rather stout, distad tapering, slightly curved prefemoral trichostele (pt) carrying a short seta, ca. 2/3× as long as femur. The latter on caudal face with a prominent, stout, finger-shaped, gently tapering distad, distomesal femoral process (fp) devoid of a trichostele, produced apically to ca. 3/4 tibia. Tibia elongate, gently tapering distad and curved apically basad towards process on femur, with an evident, caudad curved, caudal tibial process (tp). Tarsus smallest, subcylindrical, moderately sigmoid, strongly curved, narrowly rounded apically.

##### Remarks.

Unfortunately, the claw on the male legs 18 tarsus could not be observed, being broken off in both available male specimens, of which only one leg is available for examining the entire tarsus. Consequently, additional specimens are required to determine tarsal claws.

### ﻿Key to the known species of *Hyperglomeris* based on adults, modified after Golovatch (2017)

**Table:** 

1	Body completely unpigmented, pallid	***H.depigmentata***
–	Body at least partly pigmented (Figs 1, 4)	**2**
2	Dorsum entirely blackish (except for narrow pale lateral and caudal margins of tergites) (Fig. 1)	**3**
–	Dorsum with evident pale markings (Fig. 4)	**4**
3	Caudal margins 1/3 as high as tergite height; caudal edge of anal shield evidently bisinuate medially (Figs 1C, F, 2A, B). 10+1(2) ommatidia. Tibia of telopod with a large process and a small tibial cone (tc) (Fig. 2E)	***H.bicaudata* sp. nov.**
–	Caudal margins 1/5 as high as tergite height. Caudal edge of anal shield slightly emarginate medially. 8+1 ommatidia. Tibia of telopod with two small tibial cones	***H.nigra***
4	Anal shield entirely, tergum 2 partly or entirely unpigmented; tergum 2 without fine striae, but with a conspicuous sulcus anterodorsad of schism	***H.conspicua***
–	Both anal shield and tergum 2 at least partly with dark pigment	**5**
5	Dorsum without a dark axial line, background coloration either black or red-yellow	**6**
–	Dorsum with a contrasting and brighter axial line against a darker side background (Fig. 4)	**7**
6	Telopod syncoxital lobe trapeziform and truncate, its ventral margin straight	***H.dirupta***
–	Telopod syncoxital lobe semi-circular, its ventral margin rounded	***H.lamellosa***
7	Prefemoral cone very short, less than 1/5 femur. Syncoxital horns (sh) as long as syncoxital lobe (sl)	***H.maxima***
–	Prefemoral trichostele (pt) longer than 1/2 femur. Syncoxital horns (sh) higher than syncoxital lobe (sl)	**8**
8	Collum, thoracic and anal shields mostly dark brown to blackish. Male leg-pair 18 with an evidently pronounced tubercles on syncoxite. Prefemoral trichostele (pt) of telopod longer than 1/2 femur	***H.simplex***
–	Collum, thoracic and anal shields mostly pale yellowish to brownish (Fig. 4). Leg-pair 18 with a simple, subtriangular syncoxital notch (sn) (Figs 3C, D, 5C). Prefemoral trichostele (pt) of telopod longer than 2/3 femur (Fig. 5E)	***H.inkhavilayi* sp. nov.**

### ﻿Phylogenetic analysis

The COI alignment (Table 1) was 660 bp in length and contained 23 individuals, including 21 taxa from the Glomeridae as ingroup and two taxa from the Zephroniidae as outgroup. All ten pill millipede species from seven genera of the family Glomeridae in this study were retrieved as monophyletic with strong support values (Fig. 7) (77–100% bp for ML and 0.96–1 bpp for BI). However, most relationships among these species were still unresolved (< 80% bootstrap values and < 0.95 bpp for BI).

The COI tree demonstrated that the genus *Hyperglomeris* is at least paraphyletic, because of the inclusion of *Peplomerismagna* in the same clade with *H.bicaudata* sp. nov., *Hyperglomeris* sp. 1, and *H.inkhavilayi* sp. nov., although with moderate nodal support; and the exclusion of *H.simplex*, which was placed distantly at the basal position to all Glomeridae. Each of the three *Hyperglomeris* species in this study (*H.bicaudata * sp. nov., *H.inkhavilayi* sp. nov., and *H.simplex*) was retrieved as a distinct clade/species with significant support (99–100% for ML; and 0.99 bpp for BI, except 0.58 in *H.simplex*).

The interspecific divergence based on COI uncorrected p-distance among the glomerid species in this study ranged from 8.81 to 16.45%, with an average of 13.07% (Table 2), and among *Hyperglomeris* species ranged from 8.81 to 12.48%, with an average of 11.23%. This analysis also demonstrated that the intraspecific divergence for *H.bicaudata * sp. nov. was 5.30% and for *H.inkhavilayi* sp. nov. was 0.45%.

**Table 2.** Matrix of the average uncorrected p-distance (%) based on 660-bp COI barcoding region between *Hyperglomeris* species and some related glomerid and sphaerotheriid taxa. Interspecific divergence is below diagonal and intraspecific divergence is in bold.

**Taxa**	**1.**	**2.**	**3.**	**4.**	**5.**	**6.**	**7.**	**8.**	**9.**	**10.**	**11.**	**12.**
1. *Hyperglomerisbicaudata* sp. nov.	**5.30 **±** 0.85**											
2. *Hyperglomerisinkhavilayi* sp. nov.	10.76 ± 1.14	**0.45 **±** 0.27**										
3. *Hyperglomeris* sp. 1	11.93 ± 1.21	8.81 ± 1.10	**n/a**									
4. *Hyperglomerissimplex*	12.48 ± 1.15	11.40 ± 1.20	11.96 ± 1.24	**4.86 **±** 0.84**								
5. *Peplomerismagna*	10.87 ± 1.16	10.17 ± 1.16	10.01 ± 4.21	12.47 ± 1.25	**0.47 **±** 0.28**							
6. *Hyleoglomerislobus*	12.89 ± 1.22	11.65 ± 1.21	13.85 ± 1.31	11.98 ± 1.17	12.73 ± 1.27	**3.24 **±** 0.55**						
7. *Tonkinomerisnapoensis*	12.76 ± 1.17	12.00 ± 1.22	14.25 ± 1.36	13.43 ± 1.24	12.91 ± 1.30	13.58 ± 1.27	**2.16 **±** 0.57**					
8. *Rhopalomerissauda*	13.84 ± 1.22	11.99 ± 1.15	13.53 ± 1.23	12.15 ± 1.11	12.52 ± 1.15	12.25 ± 1.12	13.16 ± 1.17	**6.84 **±** 0.69**				
9. *Trachysphaeracostata*	14.46 ± 1.45	13.35 ± 1.41	14.46 ± 1.51	13.44 ± 1.36	13.86 ± 1.45	13.10 ± 1.35	14.29 ± 1.45	14.16 ± 1.30	**n/a**			
10. *Glomerismarginata*	16.45 ± 1.40	14.17 ± 1.42	16.28 ± 1.48	15.50 ± 1.41	14.84 ± 1.40	15.22 ± 1.40	14.23 ± 1.33	15.06 ± 1.31	12.93 ± 1.41	**3.04 **±** 0.69**		
11. *Sphaerobelumtruncatum*	28.79 ± 1.68	28.64 ± 1.72	29.50 ± 1.82	28.43 ± 1.70	28.36 ± 1.72	29.02 ± 1.70	28.96 ± 1.73	28.64 ± 1.65	28.57 ± 1.87	29.80 ± 1.72	**n/a**	
12. *Zephronialaotica*	30.42 ± 1.77	28.91 ± 1.74	28.75 ±1.79	28.54 ± 1.77	29.63 ± 1.77	30.58 ± 1.74	31.91 ± 1.76	29.55 ± 1.72	29.13 ± 1.85	31.35 ± 1.76	22.39 ± 1.65	**n/a**

## ﻿Discussion and conclusion

**Figure 6.**
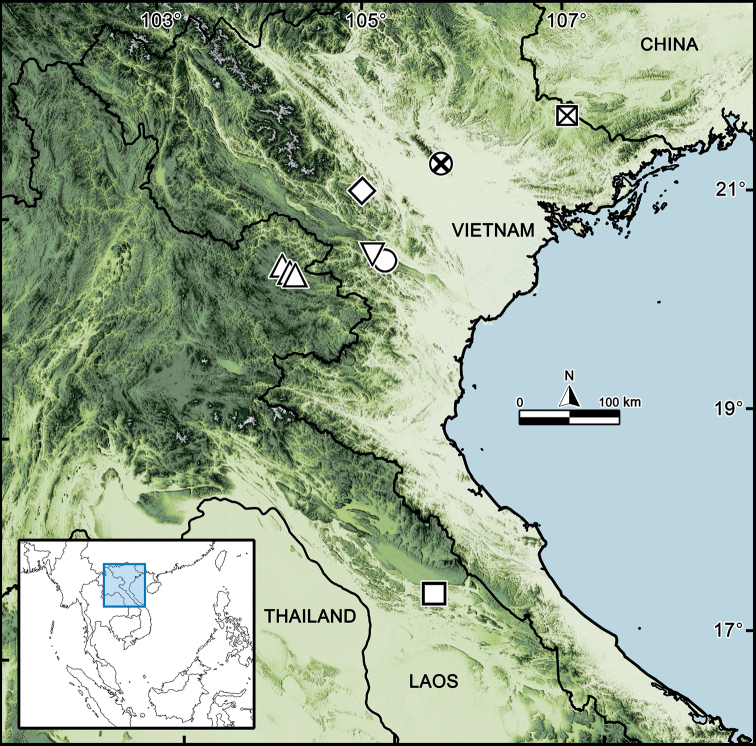
Distribution of nine *Hyperglomeris* species. **Crossed square***H.lamellosa* Silvestri, 1917 and *H.dirupta* (Silvestri, 1917) **Crossed circle***H.simplex* Nguyen, Sierwald & Marek, 2019 **Open diamond***H.nigra* Golovatch, 2017 **Inverted triangle***H.conspicua* Golovatch, 1983 and *H.maxima* Golovatch, 1983 **Circle***H.depigmentata* Golovatch, Geoffroy & VandenSpiegel, 2013 **Triangle***H.bicaudata * Likhitrakarn, sp. nov. **Square***H.inkhavilayi* Likhitrakarn, sp. nov.

This study has revealed two new species of *Hyperglomeris*, a genus new to the fauna of Laos. In addition, we have also refined the scope of the genus and the species distributions. These new records have increased the number of species of the order Glomerida in Laos from four (all in *Hyleoglomeris*) to a total of six. At present, the genus *Hyperglomeris* comprises nine species, mostly recorded from Vietnam (seven species), now also from Laos (two species) (Fig. 6). All *Hyperglomeris* species appear to be highly localized and endemic, with *H.depigmentata* probably a troglobiont, found exclusively in Hang Doi Cave. At two of these localities (Mount Mẫu Sơn and Vạn Mai), a coexistence of two species has been documented (Fig. 6). In addition to reporting these two new species, this study presents the southernmost record of *Hyperglomeris* in southern Laos. The distribution patterns (Fig. 6) clearly indicate that further new species of the genus can be expected from Laos, southern China and northern and/or eastern Thailand in the future.

The interspecific COI uncorrected p-distances among *Hyperglomeris* species in this study (8.81–16.45%) are comparable to those of European *Glomeris* species (11.5–17.1%; Wesener 2015b) and among the Vietnamese glomeridan genera (*Peplomeris*, *Hyperglomeris*, *Hyleoglomeris*, and *Tonkinomeris*) (13–15.8%; Nguyen et al. 2021). The smallest difference was retrieved between *H.inkhavilayi* sp. nov. and *Hyperglomeris* sp. 1 (8.81%), and the distance between these two species’ localities is around 180 kilometers. Thus, it is reasonable to believe that they are highly close congeners. Likewise, this result is consistent with the re-calculated interspecific distances between European *Glomeris* species, which ranged from 6.7% to 15.9%, and where the least distance (6.7–9.0%) was between *G.primordialis* and *G.klugii* (Wesener and Conrad 2016).

The intraspecific distance within the new species ranged from 0.45 to 5.3%. This is comparable to the range of the Vietnamese glomerid species, *Peplomerismagna* (0.2%) and *Rhopalomerissauda* (7.7%) (Nguyen et al. 2021). The mean intraspecific distance of *R.sauda* was rather high (6.84%) because of its extensive distribution (Nguyen et al. 2021), and the fact that the analyzed samples were gathered from multiple localities, whereas for the other species, a single locale was selected. Similarly, the relatively high value of *H.bicaudata * sp. nov. (5.3%) was obtained from samples collected from two sites for analysis; thus, there are greater differences between the two population groups compared to *H.inkhavilayi* sp. nov., which had a low value (0.45%) due to the selection of study specimens from a single population.

**Figure 7.**
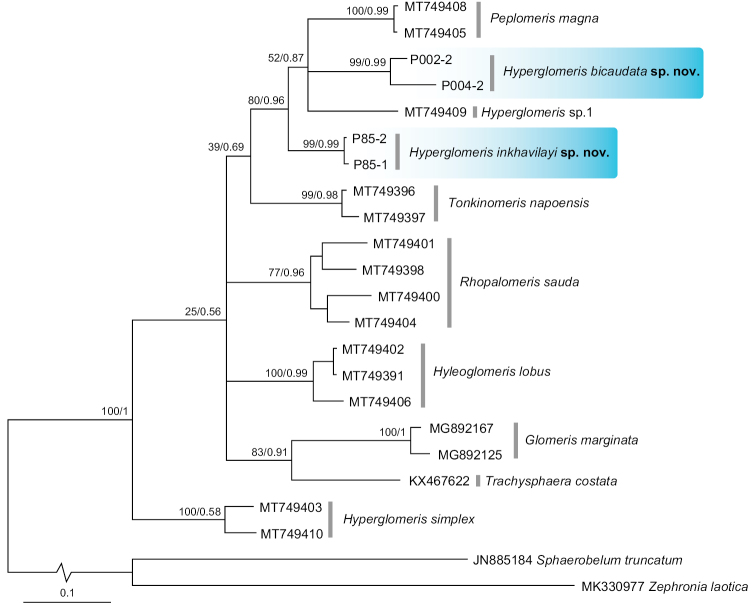
Maximum likelihood tree (ML) of pill millipedes in the family Glomeridae based on 660 bp of COI gene. Clades of new species in this study are highlighted in blue. Numbers on nodes are bootstrap values (bs) from ML analysis and bipartition posterior probability (bpp) from Bayesian inference analysis (BI), and are shown as ML/BI.

The COI tree clustered both new species with *Hyperglomeris* sp. 1 and *Peplomerismagna* with a supported clade (Fig. 7). *Peplomeris* Silvestri, 1917 and *Hyperglomeris* are closely related genera found in the same country and classified in the same subfamily Haploglomerinae. Morphological characteristics of the genus *Peplomeris* are extremely similar to those of the genus *Hyperglomeris*, including the basic structure of posterior telopods with reduced or eliminated prefemoral and femoral trichosteles. However, *Hyperglomeris* has only four apical cones on their antennae, whereas *Peplomeris* has numerous apical cones (Wesener 2015a; Nguyen et al 2019). Consequently, it is not surprising that the genetic relationship between the members in these two genera is very close. Despite this, it is premature to make conclusions about the relationships among the two genera based on the results of this study; additional persuasive evidence (i.e., more taxa and genetic markers) is needed to clarify the taxonomic status of both genera.

DNA sequence analysis was previously introduced and has provided a more reliable identification of glomerid species (Nguyen et al. 2019, 2021; Liu and Golovatch 2020). The present paper provides a phylogenetic analysis of ten members of seven genera within the family Glomeridae, including three new sequences from two recently discovered species. Unfortunately, the phylogenetic relationships among these genera is still not supported, which is consistent with studies by Liu and Golovatch (2020) and Nguyen et al. (2021). Hence, data from the COI gene alone are not sufficient to confirm the relationship between genera within this millipede family. We recommend including more genes such as 16S, 28S ribosomal RNA or other advanced molecular techniques (i.e., transcriptomic and phylogenomic data) in future studies to clarify phylogenetic relationships (Means et al. 2021; Benavides et al. 2023). Nonetheless, our findings regarding *Hyperglomeris* demonstrate that the sequencing of the COI gene is still beneficial for species delimitation and facilitates accurate identification among glomerid species.

## Supplementary Material

XML Treatment for Hyperglomeris

XML Treatment for Hyperglomeris lamellosa

XML Treatment for Hyperglomeris dirupta

XML Treatment for Hyperglomeris conspicua

XML Treatment for Hyperglomeris maxima

XML Treatment for Hyperglomeris depigmentata

XML Treatment for Hyperglomeris nigra

XML Treatment for Hyperglomeris simplex

XML Treatment for Hyperglomeris bicaudata

XML Treatment for Hyperglomeris inkhavilayi
